# Behavior Change Effectiveness Using Nutrition Apps in People With Chronic Diseases: Scoping Review

**DOI:** 10.2196/41235

**Published:** 2023-01-13

**Authors:** Emily Salas-Groves, Shannon Galyean, Michelle Alcorn, Allison Childress

**Affiliations:** 1 Department of Nutritional Sciences Texas Tech University Lubbock, TX United States; 2 Department of Hospitality & Retail Management Texas Tech University Lubbock, TX United States

**Keywords:** mobile apps, apps, mobile health, mHealth, eHealth, nutrition education, cancer, obesity, diabetes, cardiovascular disease, mobile phone

## Abstract

**Background:**

Cardiovascular disease, cancer, diabetes mellitus, and obesity are common chronic diseases, and their prevalence is reaching an epidemic level worldwide. As the impact of chronic diseases continues to increase, finding strategies to improve care, access to care, and patient empowerment becomes increasingly essential. Health care providers use mobile health (mHealth) to access clinical information, collaborate with care teams, communicate over long distances with patients, and facilitate real-time monitoring and interventions. However, these apps focus on improving general health care concerns, with limited apps focusing on specific chronic diseases and the nutrition involved in the disease state. Hence, available evidence on the effectiveness of mHealth apps toward behavior change to improve chronic disease outcomes is limited.

**Objective:**

The objective of this scoping review was to provide an overview of behavior change effectiveness using mHealth nutrition interventions in people with chronic diseases (ie, cardiovascular disease, diabetes mellitus, cancer, and obesity). We further evaluated the behavior change techniques and theories or models used for behavior change, if any.

**Methods:**

A scoping review was conducted through a systematic literature search in the MEDLINE, EBSCO, PubMed, ScienceDirect, and Scopus databases. Studies were excluded from the review if they did not involve an app or nutrition intervention, were written in a language other than English, were duplicates from other database searches, or were literature reviews. Following the PRISMA (Preferred Reporting Items for Systematic Reviews and Meta-Analyses) 2020 guidelines, the systematic review process included 4 steps: identification of records through the database search, screening of duplicate and excluded records, eligibility assessment of full-text records, and final analysis of included records.

**Results:**

In total, 46 studies comprising 256,430 patients were included. There was diversity in the chronic disease state, study design, number of participants, in-app features, behavior change techniques, and behavior models used in the studies. In addition, our review found that less than half (19/46, 41%) of the studies based their nutrition apps on a behavioral theory or its constructs. Of the 46 studies, 11 (24%) measured maintenance of health behavior change, of which 7 (64%) sustained behavior change for approximately 6 to 12 months and 4 (36%) showed a decline in behavior change or discontinued app use.

**Conclusions:**

The results suggest that mHealth apps involving nutrition can significantly improve health outcomes in people with chronic diseases. Tailoring nutrition apps to specific populations is recommended for effective behavior change and improvement of health outcomes. In addition, some studies (7/46, 15%) showed sustained health behavior change, and some (4/46, 9%) showed a decline in the use of nutrition apps. These results indicate a need for further investigation on the sustainability of the health behavior change effectiveness of disease-specific nutrition apps.

## Introduction

### Background

Cardiovascular disease (CVD), cancer, diabetes mellitus (DM), and obesity are common chronic diseases [1], and their prevalence is reaching a substantial epidemic level internationally [2]. Chronic diseases are defined by the Centers for Disease Control and Prevention broadly as “conditions that last one year or more and require ongoing medical attention or limit activities of daily living or both” [3]. Chronic diseases affect hospitalization, mortality rates, and people’s overall health and quality of life (QOL) [1]. For example, CVD remains the most prevalent cause of morbidity and mortality in high-income countries despite significant advances in treatment over the last 5 decades. Recent epidemiological data show that CVD mortality is no longer declining and is indeed rising in some communities [4], and hospitalization rates are universally increasing [5]. Furthermore, chronic conditions such as cancer, CVD, DM, and chronic respiratory diseases caused approximately 33.2 million deaths worldwide in 2019 [6]. The prevalence of obesity has increased in all World Health Organization regions since 2000, which affects other chronic conditions as it is a risk factor for the development of CVD, DM, and several cancers [6].

As the impact of chronic diseases continues to increase, finding strategies to improve care, access to care, and patient empowerment becomes increasingly essential. Therefore, mobile health (mHealth) technology is rapidly gaining popularity among health care providers and consumers [7]. mHealth technology is defined as mobile devices (ie, mobile phones or monitoring devices) intended to be worn, carried, or accessed by patients or health care providers to monitor health status or improve health outcomes [8]. Among mobile devices, the most used are smartphones, with more than three-quarters of Americans having one, and at least one-third of smartphone owners use a health app [7,9]. Health care providers use mHealth to access clinical information, collaborate with care teams, communicate over long distances with patients, and facilitate real-time monitoring and interventions. These apps provide an opportunity to increase health care access for vulnerable populations [10]. Patients use mHealth to track their health data, access their clinical records, and communicate with their providers [11].

Furthermore, a meta-analysis [12] reported promising results for mHealth interventions in the improvement of patient outcomes such as body measurements (ie, weight and waist circumference), metabolic and physiological measurements (ie, blood pressure and glucose levels), adherence to and safe use of medication, physical activity performance, meal management, and awareness of health conditions and treatment options. Mobile apps that provide tools intended to facilitate nutrition care via smartphone technologies provide patients with more autonomy, thus empowering them and offsetting patient disengagement [13]. Moreover, mobile app–based interventions effectively improve diet and diet-related health outcomes, and effect sizes are comparable with those of traditional nondigital interventions [14]. For example, mHealth interventions can help improve lifestyle behaviors related to CVD [15,16]. A recent meta-analysis [17] found that using mHealth interventions for CVD was associated with improved blood pressure and hospitalization rates. In addition, mHealth apps have emerged as supportive tools in managing cancer as they reduce the financial burden, provide access to information, and facilitate communication [18]. Different studies and meta-analyses of patients with cancer have shown the benefits of mHealth, which include reducing fatigue or pain [19,20], providing distance physical activity programs [21,22], the use of social networks to improve QOL [23], and monitoring of symptoms [24]. mHealth offers improved and cost-effective care to people with type 2 DM (T2DM) through improved diabetes self-management [25,26]. These apps seem to increase the perception of self-care by contributing to better knowledge among people with T2DM [10]. Individuals with DM also become more confident in dealing with their illness, primarily because of decreased fear resulting from a lack of information [27,28]. For obesity, a systematic review [29] has found that technology-based interventions can provide a moderately effective way of facilitating lifestyle changes and weight loss. New technologies could help reduce economic costs, improve accessibility and adherence to treatment, and increase patient motivation and weight control [30].

The use of mobile apps for mHealth and of general health apps is increasing rapidly. However, these apps focus on improving general health care concerns, with limited apps focusing on specific chronic diseases and their nutritional intervention. The available evidence on the effectiveness of mHealth apps toward behavior change to improve chronic disease outcomes is also limited. Systematic reviews to date regarding nutrition apps have focused on healthy participants or examined the effects of dietary apps on diet improvement but not on chronic diseases [31]. Most reviews were inconclusive, with the authors recommending further research in this area to demonstrate possible benefits [32-34]. From a clinical point of view, it is essential to know if an app designed for chronic diseases produces behavior changes to improve an individual’s chronic disease outcomes. From an app creator’s point of view, it is crucial to see what needs to be done when developing an app directed toward people with chronic diseases to enhance the app’s effectiveness on behavior change and produce positive outcomes. The goal of this review was to define behavioral theories associated with mHealth use, evaluate behavior change techniques (BCTs), and describe the behavior change effectiveness using mHealth nutrition interventions in people with chronic diseases (ie, CVD, DM, cancer, and obesity).

### Models and Theories of Health Behavior and Clinical Interventions

Health care and self-management of chronic conditions require effort and commitment on the part of the patient to follow treatment plans. These treatments involve many behaviors, such as dietary intake, physical activity, and prescription drug use. [35]. Theories and models are used in treatment planning to understand and explain the health behavior of individuals and can help guide clinicians to develop interventions that increase the effectiveness of health behavior change. The role of behavioral theories and models in informing digital health interventions is important to support sustainable health behavior change. A brief explanation of some of the theories and models of behavior used in these studies may help with understanding their constructs [36]: (1) the transtheoretical model shows how individuals move through 6 stages of behavior change (ie, precontemplation, contemplation, preparation, action, maintenance, and termination), which can be used to support behavior change and self-efficacy for the cessation of unhealthy behaviors and encourage a healthy lifestyle; (2) Social Cognitive Theory emphasizes self-efficacy and focuses on behavior change and outcome expectations by mastering steps to behavior change, observing others who are successful, improving mood, and ultimately increasing self-efficacy for health behavior change; (3) the Health Belief Model is based on the concept of expectancy-value and constitutes an individual’s belief that their health condition is serious, their actions will reduce their risk or illness, there is a benefit to taking action for their health condition, and they have the ability to take action for health behavior change (self-efficacy); and (4) the theory of planned behavior emphasizes that motivation that is directly influenced by ability (ie, an individual’s self-efficacy or perceived control over outside factors) is key to making a health behavior change [36]. There are other theories that can be used as well. For health behavior change, there is a general acceptance of the Health Belief Model along with a focus on self-efficacy found in many behavioral models such as the Social Cognitive Theory [35]. In addition, a commonly used theory to guide lifestyle interventions is the transtheoretical model [36]. There are several theories identified in this review that are important to chronic disease research on health behavior.

## Methods

### Information Sources and Search Strategies

In April 2022, a systematic literature search was conducted in accordance with the PRISMA (Preferred Reporting Items for Systematic Reviews and Meta-Analyses) strategy for each of the following diseases: obesity, DM, CVD, and cancer [37]. The PRISMA method allows for the transparent selection and analysis of literature for inclusion. The databases used in the search were MEDLINE, EBSCO, PubMed, ScienceDirect, and Scopus. These databases were chosen based on their extensive health- and technology-related literature [38]. The search strategy was similar for all diseases. It included the following keywords searched among titles, keywords, and abstracts: (1) *mobile applications* or *apps* or *mobile apps* or *mHealth* or *eHealth* AND *nutrition* *education* AND *obesity*, (2) *mobile applications* or *apps* or *mobile apps* or *mHealth* or *eHealth* AND *nutrition* *education* AND *type 2 diabetes*, (3) *mobile applications* or *apps* or *mobile apps* or *mHealth* or *eHealth* AND *nutrition* *education* AND *cardiovascular disease*, and (4) *mobile applications* or *apps* or *mobile apps* or *mHealth* or *eHealth* AND *nutrition* *education* AND *cancer.*

### Eligibility Criteria

To summarize the evidence available on the topic, we included primary research studies involving an app and a nutrition intervention. Studies were excluded from the review if they did not involve an app or nutrition intervention, were written in a language other than English, were duplicates from other database searches, or were literature reviews.

### Procedures

**Figure 1:**
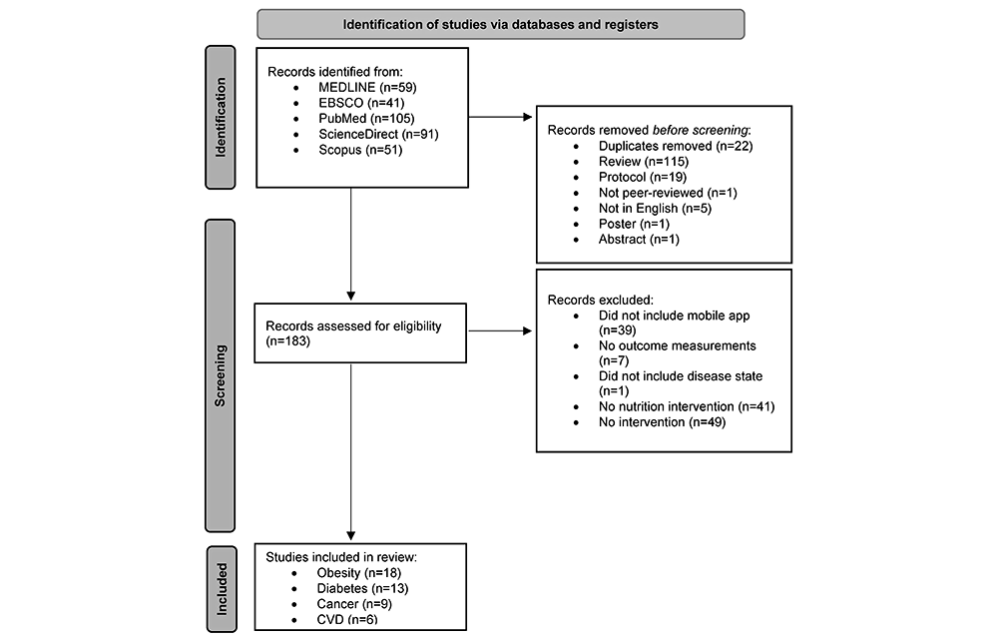
PRISMA (Preferred Reporting Items for Systematic Reviews and Meta-Analyses) flow diagram [37]. CVD: cardiovascular disease.

Following the PRISMA 2020 method, the systematic review process included four steps: (1) identification of records through the database search, (2) screening of duplicate and excluded records, (3) eligibility assessment of full-text records, and (4) final analysis of included records. This process can be seen in Figure 1. The results of the database searches were exported to Microsoft Excel (Microsoft Corp) for further analysis. Duplicates were removed. The remaining studies were moved to the eligibility phase, in which the authors assessed the eligibility of the articles based on the inclusion and exclusion criteria of the full text. The reason for exclusion was listed for each excluded article. The citation of the article, name of the mobile app used if available, type of intervention used, year of publication, population and sample details, study design details, purpose of the research, behavioral effectiveness, behavioral techniques, theory used, outcome measures, and results were recorded for all included studies. The authors independently reviewed the articles found in each search, and a second screening was performed by a different author. The process for screening was as follows: SG initially screened articles found in the obesity search, MA initially screened articles found in the DM search, ESG initially screened articles found in the cancer search, and AC initially screened articles found in the CVD search. SG performed a second screening of articles found in the DM search, MA performed a second screening of articles found in the obesity search, AC performed a second screening of articles found in the cancer search, and ESG performed a second screening of articles found in the CVD search.

## Results

### CVD Results

#### Demographic, Participant, and Study Design Details

There were 6 studies and mHealth app nutrition interventions comprising 451 patients included for CVD. Of the 6 studies, 4 (67%) randomized controlled trials, 1 (17%) qualitative descriptive design study, and 1 (17%) intervention evaluation were analyzed. The populations analyzed had diverse ethnicities, education levels, ages, and diagnoses. Furthermore, there were different diets or treatments among the studies, with variations in targeted behavioral domains and outcomes measured in the mHealth app nutrition interventions. App features for these studies included food tracking [8,39-41], physical activity tracking [8,41,42], nutrition and exercise knowledge and recommendations [8,40-43], scheduled reminders [39,40,42,43], clinician portals [41-43], connectivity to digital health devices [42], demonstration of the desired behavior [40,43], and challenges [41]. The aim of all studies (6/6, 100%) was to evaluate the effectiveness of mHealth in the CVD population. The included studies on nutrition apps for people with CVD can be found in Table 1.

**Table 1:** Included studies on cardiovascular disease (CVD; n=6).

Study	Country	Study design	Purpose	Name of mobile app and features	Behavior change theory or model	Results
Eyles et al [39]	New Zealand	Randomized controlled trial	Evaluate whether the SaltSwitch app is effective in helping people with CVD select lower-salt food purchases	SaltSwitch—all-smartphone app; features: food tracking and reminders	None	A significant reduction in mean household purchases of salt was observed during the 4-week intervention phase. A total of 87% of the participants reported using the SaltSwitch app, and 75% reported finding the SaltSwitch app very easy to use.
Indraratna et al [42]	New Zealand	Randomized controlled trial	Investigate the feasibility, efficacy, and cost-effectiveness of a smartphone app–based model of care in patients discharged after ACS^a^ or HF^b^ admission	TeleClinical Care TCC—all-smartphone app; features: physical activity tracking, knowledge, reminders, connectivity to digital health devices, and clinician portal	None	The intervention was associated with a significant reduction in unplanned hospital readmissions, including cardiac readmissions, and higher rates of cardiac rehabilitation completion and medication adherence. The average usability rating for the app was 4.5/5.
Agher et al [8]	France	Survey	Design an mHealth^c^ app, Prevent Connect, to assess its quality for evaluating patient behavior for 4 cardiovascular risk factors (unhealthy eating, sedentary lifestyle, and alcohol and tobacco consumption) and suggest personalized recommendations and mHealth interventions for risky behaviors	Prevent Connect—all-smartphone app; features: food and physical activity tracking and knowledge	None	The app was deemed of good quality, with a mean uMARS^d^ quality score of 4 on a 5-point Likert scale. The functionality and information content of the app were particularly appreciated, with a mean uMARS score of >4. The esthetics and engagement of the app were appreciated (uMARS score of 3.7). A total of 80% (42/52) of the participants declared that the app helped them become aware of the importance of addressing health behavior, and 65% (34/52) said that the app helped motivate them to change lifestyle habits.
Schmaderer et al [43]	United States	Qualitative analysis	Explore the experience of using a self-management mHealth intervention in individuals with HF to inform a future mHealth intervention study	Play-It Health—all-smartphone app; features: knowledge, reminders, clinician portal, and demonstration of behavior	None	Participants reported an overall positive experience. The education provided during the study increased self-awareness and promoted self-management of their HF. The mHealth app supported patient empowerment, resulting in better HF management and improved quality of life.
Steinberg et al [40]	United States	Randomized controlled trial	Improve adherence to the DASH^e^ diet among women with HTN^f^ or pre-HTN	Nutritionix—all-smartphone app; features: food tracking, knowledge, reminders, and demonstration of behavior	Behavior change principles	Intervention participants had lower systolic and diastolic BP^g^ compared with active comparator participants. Most intervention participants (23/29, 79%) said that they would recommend the DASH Cloud intervention to a friend or family member. However, only 34% (10/59) indicated that the feedback SMS text messages helped them reach their diet goals.
Choi et al [41]	United States	Randomized controlled trial	Discover whether dietary counseling supplied through a custom smartphone app results in better adherence to a Mediterranean diet in a non-Mediterranean population than the traditional standard-of-care counseling	Unknown name—all-smartphone app; features: food and physical activity tracking, knowledge, clinician portal, and challenges	None	There were no significant differences between EXP^h^ and SOC^i^ regarding BP, lipid parameters, HbA_1c_, or C-reactive protein. EXP achieved a significantly greater weight loss on average of 3.3 lbs versus 3.1 lbs for SOC. Adherence to the Mediterranean diet increased significantly over time for both groups, but there was no significant difference between the groups. Similarly, there was no significant difference in diet satisfaction between EXP and SOC, although diet satisfaction increased significantly over time for both groups. The proportion of participants with high Mediterranean diet compliance increased significantly over time—from 18.4% to 57.1% for SOC and from 27.5% to 64.7% for EXP; however, there was no significant difference between the groups.

^a^ACS: acute coronary syndrome.

^b^HF: heart failure.

^c^mHealth: mobile health.

^d^uMARS: User Version of the Mobile App Rating Scale.

^e^DASH: Dietary Approaches to Stop Hypertension.

^f^HTN: hypertension.

^g^BP: blood pressure.

^h^EXP: experimental.

^i^SOC: standard of care.

#### Targeted Behavior and Outcome Measures

The most commonly measured outcome in the CVD studies was usability or engagement in 83% (5/6) of the studies, followed by diet intake or quality and metabolic and physiological measurements (ie, blood pressure and urinary sodium) in 67% (4/6) of the studies. In addition, treatment adherence (ie, diet, cardiac rehabilitation, and medication) was measured in 50% (3/6) of the studies. Other measured outcomes included physical activity, weight loss, dietary knowledge, sustainability, tobacco or alcohol consumption, hospital readmissions, cost-effectiveness, food purchases, and self-management in 17% (1/6) of the studies.

#### BCTs Used

The most commonly used BCT was motivation or encouragement, which was used in all reviewed studies (6/6, 100%). Knowledge or education and self-monitoring were used in 83% (5/6) of the studies. Both prompts or cues and feedback were used in 50% (3/6) and 33% (2/6) of the studies, respectively. The least common BCTs included graded tasks or challenges, demonstration of behavior, self-efficacy, and goal setting in 17% (1/6) of the studies reviewed.

#### Behavioral Theory or Model

In total, 17% (1/6) of the analyzed studies were based on behavioral theories or models. The others (5/6, 83%) did not mention a theory. The transtheoretical model, Social Cognitive Theory, the theory of planned behavior, the Health Belief Model, the precaution adoption model, and goal-setting theories were used as the basis for 17% (1/6) of the interventions in this review section. Evidence for informing digital technology interventions reveals that the Health Belief Model has been widely used for goal setting and lifestyle changes for reducing cardiovascular risk as it focuses on confidence in one’s ability to take action [36].

#### Behavior Change Effectiveness

CVD-specific mHealth apps significantly improved the completion of cardiac rehabilitation, were significantly cost-effective, and resulted in substantial weight loss and less salt purchases in 17% (1/6) of the studies. Furthermore, significant engagement was observed in 67% (4/6) of the studies. In addition, some studies (1/6, 17%) showed improvement in blood pressure and self-management and rated the app quality as good. However, linking BCTs and theoretical frameworks to behavior change and CVD health measures is challenging. Therefore, this area of study should be investigated further.

### Cancer Results

#### Demographic, Participant, and Study Design Details

In total, 9 cancer-related studies comprising 645 patients were included. Of the 9 studies, 3 (33%) focused on survivors of breast cancer, 2 (22%) focused on breast cancer, 2 (22%) focused on esophageal cancer, 1 (11%) focused on patients with either gastric or colon cancer, and 1 (11%) focused on pancreatic cancer. Various study designs were used: 22% (2/9) prospective quasi-experimental studies, 22% (2/9) prospective pilot studies, 11% (1/9) interventional observation studies, 11% (1/9) nonrandomized 2-group controlled design studies, 11% (1/9) randomized controlled trials, 11% (1/9) randomized pretest-posttest design studies, and 11% (1/9) clinical trials were analyzed. These studies can be found in Table 2. Each app had different features, including digital diaries [7,44-49], coach feedback [7,44-47,49-51], personalized physical exercise programs and nutrition plans [49,50], general nutrition and physical exercise guidelines [45,51], psychological support courses [45,51], a secure message portal to their health care teams [7,48], health knowledge education [46,48,51], and daily tasks [48,51].

**Table 2:** Included studies on cancer (n=9).

Study	Country	Study design	Purpose	Name of mobile app and features	Behavior change theory or model	Results
Stubbins et al [7]	United States	Prospective, single-arm, open-label clinical trial	To evaluate the feasibility and usability of MOCHA^a^ to improve daily accounting of PA^b^ and food intake in survivors of breast cancer and improve engagement with health practitioners and peers	MOCHA—Apple- and Android-based app; features: food and PA tracking, coach feedback, and clinician portal	None	The average number of daily uses was approximately 3.5 times per day; participants lost an average of 2 lbs. The average usability score was 77.4, which was greater than the acceptable level. More than 90% of patients found MOCHA easy to navigate, and 84% were motivated to use MOCHA daily.
Lozano-Lozano et al [49]	Spain	Prospective test-retest quasi-experimental study	To investigate the feasibility of BENECA^c^ mHealth^d^ in an ecological clinical setting with survivors of breast cancer by studying (1) its feasibility and (2) pretest-posttest differences regarding the lifestyles, QOL^e^, and PA motivation of survivors of breast cancer	BENECA mHealth—all-smartphone app; features: food and PA tracking, coach feedback, and personalized programming	Theories of learning, Goal-Setting Theory, and Social Cognitive Theory	BENECA was considered feasible by survivors of breast cancer in terms of use (76%, 58/76), adoption (69%, 80/116), and satisfaction (positive NPS^f^). BENECA mHealth improved the QOL, EAF^g^ score, and daily moderate to vigorous PA of the participants and reduced their body weight.
Lozano-Lozano et al [50]	Spain	Prospective quasi-experimental pre-post study	(1) Check whether it is feasible to find changes in inflammation biomarkers through an mHealth strategy app as a delivery mechanism of an intervention to monitor energy balance and (2) discover potential predictors of change in these markers in survivors of breast cancer	BENECA mHealth—all-smartphone app; features: food and PA tracking, coach feedback, and personalized programming	Theory of learning, Goal-Setting Theory, and Social Cognitive Theory	Differences between pre- and postassessment CRP^h^ and IL-6^i^ showed a significant decrease in both markers. Stepwise regression analyses revealed that changes in global QOL, as well as uMARS^j^ score and hormonal therapy, were possible predictors of change in CRP concentration after using the mHealth app. Participants showed moderate satisfaction with the mHealth app; high app use (mean 47.9; maximum 56 days); and moderate to low scores in general QOL, fatigue, and pain.
Cheng et al [45]	China	Prospective, single-arm, nonrandomized pilot study	To evaluate the feasibility, safety, and efficacy of a comprehensive intervention model using an mHealth system (CIMmH^k^) in patients with esophageal cancer after esophagectomy	CIMmH—web-based program; features: food and PA tracking, knowledge, and psychological support courses	Comprehensive intervention model	At the 3-month follow-up, except for pain, eating difficulty, dry mouth, and trouble with talking, all other QOL dimensions returned to the preoperative level. There were significant reductions in weight and BMI throughout the study, and no significant changes were observed for physical fitness measured by change in the 6-minute Walk Test distance between baseline and the 1-month follow-up or between baseline and the 3-month follow-up. Depressive symptoms significantly increased 1 month after surgery, whereas other psychological measures did not show relevant changes. Although there were declines in many measures 1 month after surgery, these were much improved at the 3-month follow-up, and the recovery was more profound and faster than with traditional rehabilitation programs. Participants viewed, on average, 84% (3.38/4) of the web-based video intervention content and completed, on average, 14% (3.20/23) and 34% (9.44/28) of the web-based audio and article content, respectively. Participants completed, on average, 63% (5.01/8), 100% (1/1), and 24% (10.89/46) of the web-based nutrition, physical exercise, and psychological intervention content, respectively.
Soh et al [48]	Korea	Prospective study	To develop and validate a multidisciplinary mobile care system that can provide health education and self-management features to improve multiple clinical measures for patients with advanced gastrointestinal cancer	Life Manager—all-smartphone app; features: food and PA tracking, clinician portal, knowledge, and daily tasks	None	For the gastric cancer group, the “general gastric cancer education” was the most frequently viewed (322 times), and for the colon cancer group, the “warming-up exercise” was the most viewed (340 times). Of 13 measurements taken from participants, 9 were taken offline (response rate: 52%-90.1%), and 3 were taken on the web (response rate: 17.6%-57.4%). The overall satisfaction rate among participants was favorable and ranged from 3.93 to 4.01 on a 5-point Likert scale.
Cairo et al [44]	United States	Nonrandomized 2-group controlled study design with pre-post repeated measures	To evaluate if a readily available mHealth intervention (ie, Vida) can lead to healthier lifestyle habits for survivors of breast cancer	Vida—all-smartphone app; features: food and PA tracking and coach feedback	Behavior change theory	At 6 months, more patients in the app group experienced weight loss and had a significantly greater reduction in overall BMI. The app group also demonstrated statistically significant improvements in “strenuous” PA and had significant improvements in their dietary patterns as compared with the self-guided group. The app group had greater reduction in fatigue and improvement in depression, but these changes were not statistically significant. At 12 months, none of the app users were still using the app, but many were still following their wellness plan and had maintained their weight loss.
Yang et al [47]	Korea	Prospective pilot study	To evaluate the usefulness of a health care mobile app in preventing malnutrition and excessive muscle loss in patients with esophageal cancer receiving NACRT^l^	Noom—Android- and Apple-based app; features: food and PA tracking, coach feedback, and knowledge	None	The use (or activation) of the app was noted in approximately 70% (25/36) of the patients until the end of the trial. Compared with the 1:2-matched usual care group by propensity scores balanced with their age, primary tumor location, tumor stage, pre-RT^m^ BMI, and pre-RT SMI^n^ level, 30 operable patients showed less aggravation of the prognostic nutritional index. However, there was no significant difference in the SMI change or the number of patients with excessive muscle loss. In patients with excessive muscle loss, the walk steps significantly decreased in the last 4 weeks compared with those in the first 4 weeks. Age affected the absolute number of walk steps, whereas pre-RT sarcopenia was related to the recovery of the reduced walk steps.
Keum et al [46]	Korea	Randomized controlled study	To evaluate the efficacy of a mobile app–based program, Noom, in patients receiving chemotherapy for PDAC^o^	Noom—Android- and Apple-based app; features: food and PA tracking, coach feedback, and knowledge	None	All the study participants showed a significant improvement in nutritional status according to the PG-SGA^p^ score regardless of Noom app use. Noom users showed statistically significant improvements on the global health status and QOL scales compared with non–Noom users based on the EORTC QLQ^q^. The SMI decreased in both groups during chemotherapy. The decrement was higher in the non–Noom user group than in the Noom user group, but it was not statistically significant.
Çınar et al [51]	Turkey	Single-blinded, single-centered, and randomized design	Mobile app–based training given to cope with the side effects of EHT^r^ and how managing the symptoms and the disease process will affect the QOL of women with breast cancer	Name not provided—all-smartphone app; features: coach feedback, knowledge, psychological support courses, and daily tasks	None	QOL of the treatment group after the intervention increased, and distress level was lower compared with the control group; these results were statistically significant. Most of the patients reported that the mobile app was “informative and useful.”

^a^MOCHA: Methodist Hospital Cancer Health Application.

^b^PA: physical activity.

^c^BENECA: the Energy Balance on Cancer mobile health system.

^d^mHealth: mobile health.

^e^QOL: quality of life.

^f^NPS: Net Promoter Score.

^g^EAF: Spanish self-efficacy scale for physical activity.

^h^CRP: C-reactive protein.

^i^IL-6: interleukin-6.

^j^uMARS: User Version of the Mobile App Rating Scale.

^k^CIMmH: Comprehensive Intervention Model Using a Mobile Health System.

^l^NACRT: neoadjuvant chemoradiotherapy.

^m^RT: radiotherapy.

^n^SMI: skeletal muscle index.

^o^PDAC: pancreatic ductal adenocarcinoma.

^p^PG-SGA: Patient-Generated Subjective Global Assessment.

^q^EORTC QLQ: European Organisation for Research and Treatment of Cancer Quality of Life Questionnaire.

^r^EHT: adjuvant endocrine hormonal therapy.

#### Target Behavior and Outcome Measures

The most targeted behaviors were physical activity, observed in 56% (5/9) of the included studies, and dietary behavior, observed in 78% (7/9) of the studies. The questionnaires measured both behaviors. Furthermore, QOL was measured in 67% (6/9) of the studies, followed by app use and satisfaction in 56% (5/9) of the studies. Less frequently measured outcomes included app adherence in 44% (4/9) of the studies, depression in 33% (3/9) of the studies, and fatigue in 22% (2/9) of the studies.

#### BCTs Used

Self-monitoring and feedback were the most frequently used in mHealth apps for cancer (8/9, 89% of the studies), followed by goal setting and motivation in 44% (4/9) of the studies each. However, only 22% (2/9) of the studies used knowledge.

#### Behavioral Theory or Model

Most of the analyzed studies (6/9, 67%) seemed not to be based on common theories. Only 33% (3/9) of the studies reported at least one theory. The only reported theories were Social Cognitive Theory, Goal-Setting Theory, and the comprehensive intervention model. This result is surprising given that most apps used feedback, goal setting, and motivation to conduct behavior change. Therefore, further research could use behavioral theory or models to enhance their apps for this chronic disease. However, in physical interventions (no digital technology involved), some evidence showed that the transtheoretical model combined with other models was successful in breast cancer screening behavior as it is based on the stages of behavior change and interventions can be tailored to each individual, which increases their empowerment to make change [52].

#### Behavior Change Effectiveness

Cancer-specific mHealth apps helped significantly improve QOL in 44% (4/9) of the studies, followed by changes in anthropometrics in 33% (3/9) of the studies. A total of 11% (1/9) of the studies reported an increase in QOL and a decrease in distress level. In contrast, physical activity and nutritional status were only significantly improved in 22% (2/9) of the studies. Similar to the CVD section, linking BCTs and theoretical frameworks to behavior change and cancer health measures is challenging. Therefore, this area of study should be investigated further.

### DM Results

#### Demographic, Participant, and Study Design Details

In total, 13 studies comprising 1559 patients were included. Of the 13 studies, we analyzed 10 (77%) randomized controlled trials, 1 (8%) single-arm feasibility study, 1 (8%) case report, and 1 (8%) uncontrolled study. The studies included in this review involving nutrition apps for diabetes can be found in Table 3. Overall, there was a diverse population of patients diagnosed with prediabetes (glucose: 5.55-6.94 mmol/L or 100-125 mg/dL; HbA_1c_: 39-46 mmol/mol or 5.7%-6.4%), type 1 DM, T2DM, and gestational DM (2-hour oral glucose tolerance test level of ≥9 mmol/L). The features of each app included food tracking [53-62], education, knowledge, or recommendations [54-56,59-65], physical activity tracking [54-56,59-62], weight monitoring [56,59-62], glucose monitoring [55,57,60,61,64], blood pressure monitoring [60], community support [54,59,60], feedback [54-56,59,61,62,64], health coaches [55], clinician portals [60-62], connectivity to digital health devices [56,57], scheduled reminders [56,58,59,62], gamification [59,63], and goal setting [59]. In addition, several targeted behavioral domains and outcomes were measured as a result of the mHealth app nutrition intervention. This factor revealed some interesting insights for future investigations into digital health interventions among people with DM.

**Table 3:** Included studies on diabetes (n=13).

Study	Country	Study design	Purpose	Name of mobile app and features	Behavior change theory or model	Results
Hong et al [53]	Korea	Case study	Examine the effect of a mobile app program (“Diabetes & Nutrition”) developed between 2011 and 2012 for self-management in patients with T2DM^a^ and recommend important considerations when the mobile app program is developed	Diabetes & Nutrition—all-smartphone app; features: food tracking	None	At 3 months, body weight had decreased by 4.4 kg, waist circumference had decreased by 5 cm, and HbA_1c_ level had decreased from 7.9% to 6.1%. The medication was reduced from the dose of 850 mg to 500 mg of metformin twice a day. Since then, the patient did not continue to use the “Diabetes & Nutrition” app as their level of blood glucose had stabilized and the patient felt that it was inconvenient and annoying to use the program. At 6 months, no significant change in body weight and body composition was observed in comparison with those at 3 months.
Xu et al [54]	China	2-arm RCT^b^ with TTM^c^-based social media intervention	Examine the effectiveness of a 6-month mobile-based intervention (DHealthBar, a WeChat applet) combined with behavioral theory compared with a printed intervention in improving dietary behaviors, physical activity, and intention to change these behaviors among populations at high risk of T2DM	DHealthBar—all-smartphone app; features: food and physical activity tracking, knowledge, community support, and coach feedback	TTM	Participants in both groups reported a statistically significant decrease in energy intake at the 2 follow-up assessments compared with baseline. At 6 months, a significantly larger decrease was observed in the intervention group in energy, fat, and carbohydrate intake accompanied by a significantly larger increase in moderate-intensity physical activity compared with the control group. After 6 months of the intervention, participants were more likely to be at higher stages of dietary behaviors and physical activity than the control group.
Koot et al [55]	Singapore	6-month (24-week), single-arm, preintervention (baseline) and follow-up evaluation	Using the RE-AIM^d^ evaluation framework, this study assessed the potential effectiveness and feasibility of GlycoLeap, a mobile lifestyle management program for people with T2DM, as an add-on to standard care.	GlycoLeap—all-smartphone app; features: food and physical activity tracking, knowledge, glucose monitoring, and coach feedback	Several theoretical frameworks, including the TTM and Health Belief Model	Program engagement (implementation) started out high but decreased with time for all evaluated components. Self-reported survey data suggest that participants monitored their blood glucose on more days in the previous week at follow-up compared with baseline and reported positive changes to their diet because of app engagement. Statistically significant improvements were observed for HbA_1c_, with greater improvements for those who logged their weight more often. Participants had a 2.3% reduction in baseline weight. User satisfaction was high, with 74% (59/80) and 79% (63/80) of participants rating the app as good or very good and claiming that they would probably or definitely recommend it to others.
Griauzde et al [56]	United States	12-week, parallel, 3-arm, mixed methods pilot RCT	Examine the feasibility and acceptability of an mHealth^d^ intervention designed to increase autonomous motivation and healthy behaviors among adults with prediabetes who had previously declined participation in a diabetes prevention program; in addition, the study aimed to examine changes in autonomous motivation among adults who were offered 2 versions of the mHealth program compared with an information-only control group	mHealth—all-smartphone app; features: food and physical activity tracking, knowledge, weight monitoring, coach feedback, connectivity to digital health devices, and reminders	Self-determination theory	No significant differences were observed in adherence rates between app-only and app-plus participants. Among all participants, mean autonomous motivation measures were relatively high at baseline (6.0 out of a 7.0 scale), with no statistically significant within- or between-group differences in follow-up scores.
Torbjørnsen et al [57]	Norway	3-armed RCT with 2 intervention groups and 1 control group	This study aimed to explore associations between the level of acceptability of a mobile diabetes app and the initial ability to self-manage in patients with T2DM.	Diabetes diary app (no name)—all-smartphone app; features: food tracking, glucose monitoring, and connectivity to digital health devices	None	The study found statistically significant associations between 5 of the 8 self-management domains and “perceived benefit,” being one of the acceptability factors. However, when adjusting for age, gender, and frequency of use, only 1 domain, “skill and technique acquisition,” remained independently associated with “perceived benefit.” Frequency of use of the app was the factor that revealed the strongest association with the acceptability domain “perceived benefit.” Moreover, an association was revealed between gender and frequency of use where 69% (25/36) of the high-frequency users were men.
Alfonsi et al [58]	Canada	Iterative usability testing (3 cycles)	Test the app’s usability and potential impact on carbohydrate counting accuracy	iSpy—all-smartphone app; features: food tracking and reminders	None	Use of iSpy was associated with improved carbohydrate counting accuracy (total grams per meal), reduced frequency of individual counting errors of >10 g, and lower HbA_1c_ levels. Qualitative interviews and acceptability scale scores were positive. Moreover, 43% (9/21) of iSpy participants were still engaged, with use at least once every 2 weeks at the end of the study.
Block et al [59]	United States	Clinical trial	The aim was to evaluate the effectiveness of a fully automated, algorithm-driven behavioral intervention for diabetes prevention, Alive-PD, delivered via the internet, mobile phone, and automated phone calls.	Alive-PD—web-based application; features: food and physical activity tracking, knowledge, weight monitoring, community support, coach feedback, reminders, gamification, and goal setting	Learning theory, Social Cognitive Theory, and theory of planned behavior	Alive-PD participants achieved significantly greater reductions than controls in fasting glucose, HbA_1c_, and body weight. Reductions in BMI, waist circumference, and TG^e^ HDL^f^ ratio were also significantly greater in Alive-PD participants than in the control group. At 6 months, the Alive-PD group reduced their Framingham 8-year diabetes risk from 16% to 11%, significantly more than the control group. Participation and retention were good; intervention participants interacted with the program a median of 17 out of 24 weeks, and 71.1% (116/163) were still interacting with the program in month 6.
Koohmareh et al [63]	Iran	Interventional study	The aim of this study was to evaluate the effect of mobile game-based learning apps on improving dietary information in patients with T2DM.	Amoo—mobile phone game for all smartphones; features: knowledge and gamification	None	The results indicated a statistically significant difference between the pre- and posttest scores in the intervention group. However, there was no significant difference in fasting blood sugar.
Yu et al [60]	China	24-week, 4-arm, parallel group, nonblinded randomized trial	The aim of this study was to evaluate the effects of an MPA^g^ combined with or without SMBG^h^ on glycemic control in patients with diabetes.	Diabetes-Carer—all-smartphone app; features: food and physical activity tracking; knowledge; weight, blood pressure, and glucose monitoring; community support; and clinician portal	None	The HbA_1c_ levels in patients of all groups decreased significantly from baseline. There were significant differences in the proportions of patients that achieved HbA_1c_ <7% between groups, especially in group C and group D, compared with group A at week 24. 1,5-anhydroglucitol changes were obvious in group A and group C at week 24 from baseline. Factorial ANOVA showed that the MPA intervention was the main effective factor for HbA_1c_ change, and there was no effect on HbA_1c_ change for the SMBG intervention.
Garnweidner-Holme et al [64]	Norway	2-arm, multicenter, nonblinded RCT	The study analyzed secondary data from a 2-arm, multicenter, and nonblinded RCT to determine whether a smartphone app with targeted dietary information and blood glucose monitoring had an effect on the dietary behavior of women with GDM^i^.	Pregnant+—all-smartphone app; features: knowledge, glucose monitoring, and coach feedback	None	All the participants showed improvements in their HDS-P+^j^ from baseline. However, the Pregnant+ app did not have a significant effect on their HDS-P+. The control group reported a higher weekly frequency of choosing fish meals. No other significant differences were found between the intervention and control groups.
Agarwal et al [61]	Canada	Multicenter pragmatic RCT	The primary objective of this study was to conduct a pragmatic RCT of the Bluestar mobile app to determine if app use led to improved HbA_1c_ levels among diverse participants in real-life clinical contexts. The authors hypothesized that this mobile app would improve self-management and HbA_1c_ levels compared with controls.	BlueStar—all-smartphone app; features: food and physical activity tracking, knowledge, weight and glucose monitoring, coach feedback, and clinician portal	TTM	The results of an analysis of covariance controlling for baseline HbA_1c_ levels did not show evidence of intervention impact on HbA_1c_ levels at 3 months. Similarly, there was no intervention effect on secondary outcomes measuring diabetes self-efficacy, quality of life, and health care use behaviors. An exploratory analysis of 57 ITG^k^ participants investigating the impact of app use on HbA_1c_ levels showed that each additional day of app use corresponded with a 0.016-point decrease in participants’ 3-month HbA_1c_ levels. App use varied significantly by site as participants from one site logged in to the app a median of 36 days over 14 weeks; those at another site used the app significantly less.
Lim et al [62]	Singapore	Randomized clinical trial conducted at multiple primary care centers	Compare the effects of a culturally contextualized smartphone-based intervention with usual care on weight and metabolic outcomes	Nutritionist Buddy Diabetes—all-smartphone app; features: food and physical activity tracking, knowledge, weight monitoring, coach feedback, clinician portal, and reminders	Several theoretical models combined that included accountability, communication, and motivation to help adherence	Compared with the control group, intervention participants achieved significantly greater reductions in weight and HbA_1c_ levels, with a greater proportion experiencing a reduction in diabetes medications at 6 months. The intervention led to a greater HbA_1c_ reduction among participants with HbA_1c_ levels of ≥8%. Intergroup differences favoring the intervention were also noted for fasting blood glucose, diastolic blood pressure, and dietary changes.
Juan et al [65]	Spain	Uncontrolled study	Users would have a statistically significant increase in knowledge about the carbohydrate choices of real packaged foods after using the app.	Augmented Reality—all-smartphone app; features: knowledge	None	The results reported that their initial knowledge about carbohydrate choices was very low. This indicates that education about nutritional information in packaged foods is needed. An analysis of the pre- and postknowledge questionnaires showed that users had a statistically significant increase in knowledge of carbohydrate choices after using the app. Gender and age did not influence the knowledge acquired. The participants were highly satisfied with the app.

^a^T2DM: type 2 diabetes mellitus.

^b^RCT: randomized controlled trial.

^c^TTM: transtheoretical model.

^d^RE-AIM: Reach, Effectiveness, Adoption, Implementation, and Maintenance.

^e^TG: triglyceride.

^f^HDL: high-density lipoprotein.

^g^MPA: mobile phone app.

^h^SMBG: self-monitoring of blood glucose.

^i^GDM: gestational diabetes mellitus.

^j^HDS-P+: healthy dietary score for Pregnant+.

^k^ITG: immediate treatment group.

#### Targeted Behavior and Outcome Measures

The most frequently targeted behaviors were glycemic control measured using HbA_1c_ and DM self-efficacy or self-management in 46% (6/13) of the reviewed studies. In addition, dietary behavior, levels of engagement, user acceptability, or motivation, and weight or BMI were measured in 46% (6/13) of the studies, followed by waist circumference in 23% (3/13) of the studies. Less frequently measured outcomes included physical activity, stages of change, carbohydrate counting accuracy, QOL, health care use behavior, and lipids in 23% (3/13) of the studies.

#### BCTs Used

Numerous BCTs can be used to induce behavior change. All the DM studies reviewed (13/13, 100%) used knowledge and education, followed by self-monitoring in 77% (10/13) of the studies. Both social support or encouragement and autonomous personalized feedback were used in 54% (7/13) of the studies. Prompts or cues were used in 31% (4/13) of the studies, followed by graded tasks and gamification in 15% (2/13) of the studies. Real-time feedback and goal setting were used in 15% (2/13) of the studies reviewed. Unfortunately, no standard definition of techniques is included in the BCTs, making it challenging to identify the techniques used in the interventions [66].

#### Behavioral Theory or Model

Some of the analyzed studies (7/13, 54%) seemed not to be based on behavioral theories or models. In total, 46% (6/13) of the studies reported at least one theory. The transtheoretical model was most frequently used in 23% (3/13) of the studies, followed by Social Cognitive Theory, self-determination theory, models centering on cues and triggers, theory of planned behavior, behavioral economics, positive psychology, and motivational interviewing as other reported theories. Some research has shown that Social Cognitive Theory has been used in feasibility studies among populations with diabetes as it can increase confidence and promote greater sustained effort to change, making it a guide for digital technology interventions. This theory also includes skill training, which can benefit diabetes management and education programs [67,68].

#### Behavior Change Effectiveness

DM-specific mHealth apps improved glycemic control by significantly reducing HbA_1c_ values in 46% (6/13) of the studies. In addition, 15% (2/13) of the interventions [53,62] resulted in a decrease in the medications used for glycemic control. Some studies (6/13, 46%) showed significant engagement; however, 17% (1/6) of these studies showed a decline in engagement over time, and 17% (1/6) did not have follow-up data for engagement. Sustainability needs to be considered for the effectiveness of these types of interventions. There were also significant changes in anthropometrics in 31% (4/13) of the reviewed studies. DM self-efficacy and self-management, decrease in energy intake, and increase in physical activity were observed in 8% (1/13) of the studies. Not all studies analyzed the same outcomes for each intervention, making it difficult to link BCTs and theoretical frameworks to behavior change and health measures.

### Obesity Results

#### Demographic, Participant, and Study Design Details

A total of 18 studies comprising 253,775 patients were included. Among these 18 studies, we analyzed 5 (28%) randomized controlled trials, 5 (28%) experimental studies, 3 (17%) feasibility studies, 2 (11%) observations, and 1 (6%) prospective cohort study. The studies involving nutrition apps among people with obesity can be found in Table 4. The studies focused on obesity prevention and treatment in many diverse populations that ranged in age, socioeconomic background, and physical status (ie, pregnancy and post partum). App features included physical activity tracking [69-78], food tracking [70,72-83], knowledge, education, or recommendations [70,72-76,78,79,82-86], push notifications or scheduled reminders [74-76,78,79,81-84,86], weight monitoring [75,76,78,81,85,86], behavior demonstration [69,75,83], motivational challenges [69,75-78,83], goal setting [69,73-75,78,81,84,85], feedback [69,70,73,75,76,81-83,86], community support [70,75,78,85], connectivity to digital health devices [70,72,81,86], clinician portals [72,73,82], and access to a health coach [76-78,81]. Furthermore, numerous behaviors were targeted, and the outcomes were analyzed to determine the effectiveness of a mobile nutrition intervention in promoting healthy weight. This information will help develop approaches and techniques for digital health behavior change interventions to prevent and treat obesity.

**Table 4:** Included studies on obesity (n=18).

Study	Country	Study design	Purpose	Name of mobile app and features	Behavior change theory or model	Results
Lubans et al [69]	Australia	Cluster RCT^a^	Focused on the promotion of lifetime (eg, resistance training) and lifestyle (eg, active transport) physical activities and was aligned with current physical activity guidelines, which include a recommendation to engage in muscle- and bone-strengthening physical activities at least 3 days per week	ATLAS^b^—all-smartphone app; features: physical activity tracking, behavior demonstration, challenges, goal setting, and coach feedback	Self-determination theory and Social Cognitive Theory	Focus group participants reported enjoyment of the program and felt that it had provided them with new skills, techniques, and routines for the future. However, their engagement with the smartphone app was limited. Barriers to the implementation and evaluation of the app included limited access to smartphone devices, technical problems with the push notifications, lack of access to use data, and the challenges of maintaining participants’ interest in using the app.
Griffin et al [84]	United States	1-group, pre- to posttest study design	To evaluate changes in dietary and physical activity behaviors and weight after implementation of a 12-week SMS text messaging initiative (My Quest)	SMS text messaging initiative (My Quest)—all smartphones; features: knowledge, reminders, and goal setting	Social Cognitive Theory	Participants significantly improved dietary and physical activity behaviors and food environment, increased their dietary and physical activity goal setting, and reduced their body weight. A total of 56 posttest assessments were completed (84% response rate).
Van der Pligt et al [71]	Australia	Pilot intervention study nested within a cluster RCT	Effectiveness of the Mums OnLiNE^c^ intervention with respect to reducing PPWR^d^ and improving diet, physical activity, and sedentary behavior	Mums OnLiNE Combined and InFANT Extend^e^—web-based application or smartphone app with telephone-based support; features: physical activity tracking	Social Cognitive Theory	Mean PPWR decreased in the intervention group and the comparison group 2 although the changes were not significant. Mean waist circumference for all groups exceeded recommendations at baseline but decreased to below recommendations for women in the intervention group and significantly for the intervention group compared with comparison groups 1 and 2. Changes in diet, physical activity, or sedentary behaviors were not significant.
Hull et al [79]	United States	Observational design based on data collected after the testing period	This paper describes the development and beta testing of the CHEW^f^ smartphone app. The objective of beta testing was to test the CHEW app prototype with target users focusing on use, usability, and perceived barriers and benefits of the app.	CHEW smartphone app—Android-based app; features: food tracking and knowledge	Socioecological model	Study participants used the app on average once a week for approximately 4.5 minutes per session. Use of specific features averaged at 1-2 times per month for shopping-related activities and 2-4 times per month for the snack gallery. Mothers classified as users rated the app’s WIC^g^ Shopping Tools relatively high on usability and benefits. The Yummy Snack Gallery and Healthy Snacking Tips scored higher on usability than on benefits, suggesting that the nutrition education components may have been appealing.
Bughin et al [72]	France	Randomized controlled study	The aim of this study was to compare the changes in body composition, anthropometric parameters, exercise capacity, and QOL^h^ within 12 weeks of patients in the TR^i^ program with those of usual care patients with obesity.	TR Program—Android-based or web-based; features: food and physical activity tracking, knowledge, connectivity to digital health devices, and clinician portal	None	No significant group × time interaction was found for fat mass. Compared with the UCG^j^, TRG^k^ patients tended to significantly improve their waist-to-hip ratios and improved their QOL physical impact. Significant time effects were observed for body composition, 6-minute Walk Test distance, exercise metabolism, sedentary time, and QOL. Adherence (95%) and satisfaction in the TRG were good.
Toro-Ramos et al [78]	Korea	Intervention	This study investigated the efficacy of a smartphone intervention using a designated app with a lifestyle intervention–focused approach, including a human coaching element, toward weight loss in Korean adults who were overweight or obese.	Noom—Android- and Apple-based app; features: food and physical activity tracking, knowledge, reminders, weight monitoring, challenges, goal setting, community support, and coach feedback	None	Participants showed a clinically significant weight loss effect of −7.5% at the end of the 15-week program, and at a 52-week follow-up, a weight loss effect of −5.2% was maintained. At 15 weeks, percentage of body fat and visceral fat decreased by −6.0% to –5.4% and −3.4 kg to –2.7 kg, respectively. Fasting blood glucose level also decreased significantly by −5.7 to –14.6 mg/dL at 15 weeks. Lipid parameters showed significant improvements except for high-density lipoprotein cholesterol. The frequency of logging meals and exercise was associated with body fat loss.
Pellegrini et al [70]	United States	6-month technology-supported weight loss trial	Examine within-person variation in dietary self-monitoring during a 6-month technology-supported weight loss trial as a function of time-varying factors, including time in the study, day of the week, and month of the year	ENGAGED^l^—smartphone provided with the app; features: food and physical activity tracking, knowledge, feedback, community support, and connectivity to digital health devices	None	Participants recorded less as time in the study progressed. Fewer foods were reported on the weekends compared with on weekdays. More foods were self-monitored in January compared with in October; however, a seasonal effect was not observed.
Dodd et al [74]	Australia	Multicenter, nested randomized trial	The objective was to evaluate the impact of a smartphone app as an adjunct to face-to-face consultations in facilitating dietary and physical activity change among pregnant women.	Name not provided—smartphone was provided with the app; features: food and physical activity tracking, knowledge, reminders, and goal setting	None	Mean difference in HEI^m^ score was 0.01 at 28 weeks of pregnancy and −1.16 at 36 weeks of pregnancy. There was no significant additional benefit from the provision of the smartphone app in improving HEI score. Although all women improved dietary quality throughout their pregnancy, use of the smartphone app was poor.
Stasinaki et al [77]	Switzerland	Unblinded RCT	The objective of the study was to assess novel obesity management that moved the focus from on-site consultations in a specialized childhood obesity center to an appealing, youth-friendly, low-threshold mobile intervention (PathMate2) under the supervision of pediatric obesity experts.	PathMate2—smartphone provided with the app; features: food and physical activity tracking, challenges, and coach feedback	None	At intervention start, median BMI SDS^n^ of all patients was 2.61. BMI-SDS decreased significantly in the control group at time 1 but not at time 2 and did not decrease in the intervention group during the study. Muscle mass, strength, and agility improved significantly in both groups at time 2; only the intervention group significantly reduced their body fat at time 1 and time 2. Average daily PathMate2 app use rate was 71.5%. Cortisol serum levels decreased significantly after biofeedback but with no association between stress parameters and BMI-SDS.
Ali et al [73]	UAE^o^	Nonrandomized, 2-arm feasibility study	Develop and test a nutrition education intervention delivered via a website and mobile apps to university students in the UAE who were overweight and obese	Rashakaty-Basic and Rashakaty-Enhanced—web-based application and all-smartphone app; features: food and physical activity tracking, knowledge, goal setting, feedback, and clinician portal	Social Cognitive Theory	There was no significant difference in weight loss between the 2 arms. However, waist circumference decreased more in the Rashakaty-Enhanced group. Changes in knowledge related to sources of nutrients and diet-disease relationships were significantly higher among the Rashakaty-Enhanced group. Rashakaty-Enhanced participants reported increased number of days spent on moderate physical activity and minutes walked. They also reported higher scores in social support from friends to reduce fat intake and from family and friends to increase physical activity.
Senecal et al [86]	China	Retrospective observational analysis	To evaluate whether individuals following a weight loss program based on a mobile app, wireless scale, and nutritional program but no face-to-face care could achieve clinically significant weight loss in a large cohort	MetaWell—Android- and Apple-based app; features: knowledge, reminders, weight monitoring, feedback, and connectivity to digital health	None	251,718 individuals (79% female) were included with a mean weight loss of 4.3 kg and a mean follow-up of 120 days. Mean weight loss at 42, 60, 90, and 120 days was 4.1 kg, 4.9 kg, 5.6 kg, and 5.4 kg, respectively. At 120 days, 62.7% of participants had lost at least 5% of their initial weight. Both genders and all use frequencies showed statistically significant weight loss from baseline at each interval, and this loss was greater in men than in women. The frequency of recording was associated with greater weight loss when comparing high-, medium-, and low-use groups at all time intervals investigated.
Delisle Nyström et al [82]	Sweden	2-arm parallel RCT	Investigate the 12-month after-baseline measurements of the MINISTOP^p^ intervention	MINISTOP—both web-based application and all-smartphone app; features: food tracking, knowledge, reminders, feedback, and clinician portal	Social Cognitive Theory	At the 12-month follow-up, no statistically significant difference was observed between the intervention and control groups for FMI^q^, and no maintained effect for the change in composite score was observed.
Stein and Brooks [81]	United States	Longitudinal observational study	Evaluate weight loss, changes in meal quality, and app acceptability among users of the HCAI^r^ with the overarching goal of increasing access to compassionate health care via mHealth^s^	Lark Weight Loss HCAI—Android- and Apple-based app; features: food tracking, reminders, weight monitoring, goal setting, coach feedback, and connectivity to digital health devices	None	Weight loss was 2.38% of baseline weight. The average duration of app use was 15 weeks, and users averaged 103 sessions each. The percentage of healthy meals increased by 31%. The in-app user trust survey had a 100% response rate and positive results, with a satisfaction score of 87 out of 100 and Net Promoter Score of 47.
Prasad et al [80]	United States	Open-label, nonrandomized, prospective 90-day TRE^t^ intervention	The primary aim was to test the feasibility of a TRE intervention administered via a smartphone app aimed at reducing the eating window by 4 hours in individuals with a habitually prolonged eating window and determine the efficacy of a 90-day TRE intervention in reducing body weight and blood pressure in adults who were overweight and obese. A secondary aim was to monitor the adherence to the intervention over time.	MyCircadianClock—all-smartphone app; features: food tracking	None	The mean duration of the baseline eating window was 14 hours and 32 minutes (SD 2 hours and 36 minutes), with 56% of participants with a duration of ≥14 hours. TRE participants successfully decreased their eating window from 16 hours and 4 minutes (SD 1 hour and 24 minutes) to 11 hours and 54 minutes (SD 2 hours and 6 minutes) and reduced the number of daily eating occasions by half. Adherence to logging and to the reduced eating window was 64% (SD 22%) and 47% (SD 19%). TRE resulted in decreases in body weight, waist circumference, and systolic blood pressure.
Simpson et al [85]	Scotland	Feasibility RCT	To develop and assess the feasibility and acceptability of an app-, web-, and social support–based intervention in supporting adults with obesity to achieve weight loss goals	HelpMeDoIt!—all-smartphone app and web-based application; features: knowledge, reminders, weight monitoring, goal setting, and community support	Social Cognitive Theory and control theory	Of the 54 (74%) participants who downloaded the app, 48 (89%) used it. Objective physical activity measures perhaps showed the most potential (daily step count [1187 steps] and sedentary time [−60.8 min]). However, these outcomes were poorly completed.
Lin et al [75]	United States	RCT	To compare an mHealth intervention delivered via a CP^u^ app with usual care controls and compare with an in-person and phone-supplemented personal coaching intervention enhanced by CP self-monitoring with usual care	CITY^v^—all-smartphone app; features: food and physical activity tracking, knowledge, reminders, weight monitoring, behavior demonstration, challenges, goal setting, feedback, and community support	Rooted in theoretical models and behavioral framework	Use of the app was highest during month 1 for both arms; thereafter, use dropped substantially and continuously until the study end. During the first 6 months, the mean percentage of days that any app component was used was higher for the CP arm (74.2%) than for the personal coaching arm (48.9%). The CP arm used the apps an average of 5.3 times per day, whereas the personal coaching participants used them 1.7 times per day. The former self-weighed more than the latter (57.1% of days vs 32.9% of days). Furthermore, the percentage of days that any app component was used, number of app uses per day, and percentage of days self-weighed all showed significant differences across the 4 weight categories for both arms. Pearson correlation showed a negative association between weight change and the percentage of days that any app component was used, number of app uses per day, and percentage of days self-weighed.
Chew et al [76]	Singapore	Prospective single-cohort study	Assess the effectiveness of adolescent engagement with a mobile app–based lifestyle intervention program as an early intervention before enrollment in a clinic-based multidisciplinary weight management program	Kurbo—all-smartphone app; features: food and physical activity tracking, knowledge, reminders, weight monitoring, challenges, and coach feedback	None	Kurbo engagement was high, with 83% (33/40) of participants completing at least 7 coaching sessions. In total, 78% (18/23) of participants rated the app as good to excellent, and 70% (16/23) stated that they would recommend it to others. There were no statistically significant changes in BMI *z* scores at 3 or 6 months. Participants showed statistically significant improvements in measured body fat percentage, self-reported QOL, and self-reported caloric intake from the 3-day food diaries at 3 and 6 months.
Kay et al [83]	United States	Randomized controlled feasibility trial	Comparing app-based diet tracking (active comparator) with app-based diet tracking plus feedback on DASH^w^ adherence via SMS text message (intervention)	DASH Cloud—all-smartphone app; features: food tracking, knowledge, reminders, behavior demonstration, challenges, and feedback	None	DASH Cloud did not enhance DASH adherence over diet tracking alone but resulted in greater reductions in blood pressure.

^a^RCT: randomized controlled trial.

^b^ATLAS: Active Teen Leaders Avoiding Screen-Time.

^c^OnLiNE: Online, Lifestyle, Nutrition, and Exercise.

^d^PPWR: postpartum weight retention.

^e^InFANT Extend: Extended Melbourne Infant Feeding Activity and Nutrition Trial.

^f^CHEW: Children Eating Well.

^g^WIC: Women, Infants, and Children.

^h^QOL: quality of life.

^i^TR: telerehabilitation.

^j^UCG: usual care group.

^k^TRG: TR group.

^l^ENGAGED: e-Networks Guiding Adherence to Goals in Exercise and Diet.

^m^HEI: healthy eating index.

^n^SDS: SD score.

^o^UAE: United Arab Emirates.

^p^MINISTOP: Mobile-Based Intervention Intended to Stop Obesity in Preschoolers.

^q^FMI: fat mass index.

^r^HCAI: Health Coach AI.

^s^mHealth: mobile health.

^t^TRE: time-restricted eating.

^u^CP: cell phone.

^v^CITY: Cell Phone Intervention For You.

^w^DASH: Dietary Approaches to Stop Hypertension.

#### Targeted Behavior and Outcome Measures

Anthropometric measurements (ie, weight, BMI, and waist-to-hip ratio) were the most targeted health outcomes, with behavior change in 78% (14/18) of the reviewed studies, followed by diet quality and physical activity in 61% (11/18) of the studies. In addition, engagement, user acceptability, or motivation levels and body composition were measured in 39% (7/18) and 28% (5/18) of the studies, respectively. QOL, behaviors (ie, goal setting and self-efficacy), and CVD measures (ie, blood pressure, heart rate, glucose, and lipids) were included as outcomes in 22% (4/18) of the studies. Less frequent measures included stress parameters (ie, chronic stress and cortisol levels), diet knowledge, reduced screen time, and behavior and health maintenance, observed in 6% (1/18) of the studies.

#### BCTs Used

BCTs were incorporated into each intervention. Self-monitoring was the most commonly used BCT (14/18, 78% of the reviewed studies), followed by knowledge or education (12/18, 67% of the studies). Goal setting was used in 56% (10/18) of the studies, feedback and encouragement were used in 56% (10/18) of the studies, and prompts or cues and intention formation were used in 50% (9/18) of the studies. Feedback through private messages was used in 39% (7/18) of the studies, followed by community or social support and demonstration of behavior in 33% (6/18) of the studies each. Graded tasks and gamification or incentives were used in 17% (3/18) of the studies. Finally, information about health benefits and consequences was used in 6% (1/18) of the studies in this section. Clinician portals were used in 17% (3/18) of the interventions to improve patient care. Although it is not a BCT, it is worth mentioning as a vital intervention component.

#### Behavioral Theory or Model

Some interventions (10/18, 56%) did not mention the basis of behavioral theories or models. A total of 6% (1/18) of the studies mentioned that they were rooted in theoretical models but did not specify which ones. Social Cognitive Theory was the most frequently used behavior model in 33% (6/18) of the studies. Self-determination theory was used in 11% (2/18) of the studies, followed by the socioecological model, control theory, and social support theory in 6% (1/18) of the studies. Furthermore, Social Cognitive Theory has been used in feasibility studies on mHealth technology lifestyle interventions for obesity, for example, by using the principle of verbal persuasion through methods such as personalized encouragement that help individuals realize that they have the capability to make the necessary healthy lifestyle changes to lose weight [87].

#### Behavior Change Effectiveness

Obesity-specific mHealth apps improved weight through significant reductions in 39% (7/18) of the studies. Some studies (4/18, 22%) showed a substantial decrease in body fat percentage and waist circumference. There were also significant changes in cardiovascular measurements (ie, blood pressure and lipids) and QOL in 11% (2/18) of the reviewed studies. Changes in self-efficacy, decreases in energy intake and screen time, and increases in knowledge of nutrients and physical capabilities were observed in 6% (1/18) of the studies. In total, 11% (2/18) of the studies analyzed and reported significance in the maintenance of weight loss, whereas 11% (2/18) of the studies reported a decline in changes over time. The sustainability of these interventions is an area for future research to determine the effectiveness of these types of interventions.

## Discussion

### Principal Findings

Overall, mHealth apps used for various chronic disease populations did improve health. These studies showed significant improvements in QOL, cardiac rehabilitation completion, glycemic control (ie, HbA_1c_), weight reduction, and reduction in physiological measures (ie, blood pressure and lipids). In addition, some studies (3/46, 7%) showed improved self-efficacy and self-management of chronic diseases. Although many studies (35/46, 76%) did not measure long-term effectiveness, some (3/46, 7%) showed significance in maintaining weight loss, whereas others (3/46, 7%) showed a decline in changes or engagement in app use over time. A total of 24% (11/46) of the studies measured maintenance of health behavior change. Of these 11 studies, 7 (64%) sustained behavior change for approximately 6 to 12 months, and 4 (36%) showed a decline in behavior change or discontinued app use.

The main difference between the sustainability of health behavior change was the inclusion of a clinician portal or access to a health coach. Griauzde et al [56] measured postintervention qualitative data that provided reasons for app satisfaction, dissatisfaction, and ways to improve. Reasons for satisfaction included “encouraged self-reflection,” reasons for app dissatisfaction included “did not consider personal circumstances,” and strategies to improve the intervention included “increased interpersonal contact.” Of the 46 studies, 9 (20%) included a clinician portal as a feature to enhance the app intervention, allowing for additional communication between clinicians and patients. Of the 9 studies, 7 (78%) had an effective health behavior change. Silk et al [88] reported acceptability and easy-to-use features regarding the integration of the clinician portal into their mHealth intervention. This component should be considered for health behavior change interventions and investigated further.

Of the 46 nutrition apps in this review, 37 (80%) included some type of self-monitoring feature. Of those 37 apps, 35 (95%) had at least one significant improvement or good usability rating to promote engagement with mobile apps for chronic disease self-management. This is in alignment with other research that shows that the most common self-management application for mHealth is a tracking feature [89]. In addition, when surveying clinicians working in diabetes and weight management patient care settings, the most adoptable apps included self-monitoring features [90]. However, the apps that did not include self-monitoring or tracking features showed significant improvements as well. In addition, there was variability in other features combined with tracking features (ie, knowledge, goal setting, coach feedback, and clinician portals), making it difficult to attribute behavior change to self-monitoring features alone. More research is needed to correlate specific features with health behavior change.

Except for cancer populations, a key finding in this review was that 41% (19/46) of the nutrition app interventions targeted weight management, and 58% (11/19) of those studies were effective in health behavior change. This finding is similar to that of Fakih El Khoury et al [91], who reported that dietary mobile apps positively affected measured nutritional outcomes in chronic diseases, especially weight loss. The key finding for cancer populations was that mHealth nutrition apps can significantly improve QOL. A similar result was found in a review analyzing the use of mHealth involving nutrition in a chronic kidney disease population [92]. A total of 28% (13/46) of the studies examined changes in glycemic control, HbA_1c_, and other biometric measurements (ie, blood pressure and lipids). In total, 69% (9/13) of those studies showed a significant reduction in their measurements when using a nutrition app in their intervention. In addition, 15% (2/13) of those studies showed a decrease in medication treatment for glycemic control [53,62]. Eberle et al [93] concluded that mHealth apps with a nutrition intervention effectively enhanced diabetes management, which is comparable with our review results.

There was diversity in the chronic disease state; study design; number of participants; and variety of in-app features, BCTs, and behavior models used in the studies. However, linking these factors to a particular behavior change and health outcome posed to be difficult, which is not different from Taj et al [94] in their review of digital health behavior change technology. This review provides insight into the theories and theoretical frameworks or models used in nutrition-focused mHealth interventions to increase understanding and translate them into practice, which is essential for developing behavior change for the sustainability of health improvement. For example, this scoping review found that most apps used BCTs, such as self-monitoring in 80% (37/46) of the interventions, knowledge in 72% (33/46) of the interventions, feedback in 54% (25/46) of the interventions, and goal setting in 20% (9/46) of the interventions, for effective health behavior change, as another review [31] demonstrated.

In addition, our review found that less than half (19/46, 41%) of the studies based their nutrition apps on a behavioral theory or its constructs. Some studies (14/46, 30%) that based their interventions on theories had improved engagement, satisfaction, or app use among participants and suggested that incorporating a behavioral theory in mHealth interventions is an effective strategy, which is a comparable finding with that of another review [91]. However, other factors play a role in usability, acceptability, and overall user satisfaction. The app features, BCTs, and theory-based interventions will all affect the effectiveness of mHealth nutrition apps in health behavior change toward chronic diseases.

Another significant development in the sector of health technology is that smartphones can also be embedded with sensors or coupled with wearable sensors for health monitoring, which could enhance a nutrition app’s effectiveness in health behavior change. Examples of these devices that could be coupled with nutrition apps include motion sensors such as accelerometers, gyroscopes, and magnetometers that measure motion and physical activity [95]; wearable devices such as glasses (analyzing the intake pattern with smart glasses) and rings (ring-type tactile sensors to detect food mass) for food intake measurement [96]; portable and handheld single-lead electrocardiogram devices in addition to fitness trackers to measure heart rate and heart rate variability [95]; wristwatches to measure glucose levels extracted from skin interstitial fluid through reverse ionophoresis or through saliva-, sweat-, or tear-based wearable biosensors [97]; a wrist-wearable watch with the function of a pulsimeter without a cuff to measure blood pressure [97]; and ingestible capsules that can be used for medical health monitoring [98]. These enhance personal health care and performance monitoring with the potential to complement nutrition apps and have a broad impact on our society.

### Limitations

Although the results of this study showed health improvements achieved when using mHealth nutrition apps for behavior change in chronic diseases, some limitations need to be addressed. An important limitation is the lack of research on mobile apps’ long-term effects (>1 year) on disease state populations. Therefore, there is no conclusive result on their long-term behavior change to determine whether the behavior continues to improve or reverses compared with the baseline. Consequently, there need to be more long-term studies conducted. Second, many studies (24/46, 52%) did not include a control group for comparison, and their sample size was small, limiting the interpretation of the results of the studies. Third, many apps are at risk of becoming rapidly obsolete owing to the fast pace at which technologies progress and, therefore, new technological innovations must be considered [8]. For example, the latest mobile technologies can connect and interact with each other, update and track personal health data in real time, and send alerts to users [8]. Similarly, most health apps have encountered serious usability problems or have not undergone usability assessment [99]. Usability affects the efficiency and efficacy of the app (ie, time to complete tasks and errors) [8]. Usability must be considered to increase the chance of the app being successfully adopted by patients [8]. Barriers to using mHealth apps include the patient’s lack of integration of technology into everyday life [12] and difficulties using mobile apps [11]. In the older adult population, health problems such as cognitive changes related to aging, disability, and lack of confidence are reasons for not using digital technology [18,19]. Further research is needed to evaluate patients’ experiences with apps and the benefits gained as a result. There could be a slight bias from the user perspective as there were 2% (1/46) of apps that were Android-only or web-based applications. The others (45/46, 98%) were web-based applications or all-smartphone apps. There were a few apps (4/46, 9%) that were provided through the smartphone that participants were given as part of the study, and the smartphone was not specified. A few studies (4/46, 9%) did not report the data privacy rules or the effects of users and funders on the app interventions. In addition, there are important confidentiality and funding issues that must be considered when designing interventions [100]. Finally, proficient health care providers should be involved in the app development stage to address safety during self-management and health education [101]. There is a need for comprehensive, efficient, and flexible mobile apps for the self-management of disease states with more features to increase the number of long-term users and induce better self-management and patient empowerment [101,102]. We did not conduct a systematic review or meta-analysis and, thus, did not weigh the quality of evidence or study design against the reported results. Some studies (6/46, 13%) included few participants, and the diversity of study objectives, designs, and outcomes made it difficult to compare them. We reviewed the current evidence to expand the knowledge base regarding the impact of nutrition apps on chronic disease management and assess the effectiveness of health behavior change.

### Conclusions

In this scoping review, the use of mHealth nutrition apps and their effects on health behavior change were analyzed for 4 diseases (ie, cancer, CVD, DM, and obesity). The results suggest that mHealth apps involving nutrition can significantly improve health outcomes for people with chronic diseases. The study design, demographics, targeted behavior, health outcomes, BCTs, behavioral theories, and behavior change effectiveness were profoundly diverse among these studies, indicating that a *one-size-fits-all* approach for designing and implementing nutrition apps as part of chronic disease treatment is not possible. Tailoring nutrition apps to specific populations is recommended for effective behavior change and improvement of health outcomes. In addition, some studies (7/46, 15%) showed sustained health behavior change, and some (4/46, 9%) showed a decline in the use of the nutrition apps. These results indicate a need for further investigation on the sustainability of the health behavior change effectiveness of disease-specific nutrition apps.

## Abbreviations

BCT: behavior change technique
CVD: cardiovascular disease
DM: diabetes mellitus
HbA_1c_: glycated hemoglobin
mHealth: mobile health
PRISMA: Preferred Reporting Items for Systematic Reviews and Meta-Analyses
QOL: quality of life
T2DM: type 2 diabetes mellitus
